# Iberian fish records in the vertebrate collection of the Museum of Zoology of the University of Navarra

**DOI:** 10.1038/sdata.2016.91

**Published:** 2016-10-11

**Authors:** Amaia A. Rodeles, David Galicia, Rafael Miranda

**Affiliations:** 1 University of Navarra, School of Sciences, Department of Environmental Biology, Irunlarrea 1, 31080 Pamplona, Spain

**Keywords:** Ichthyology, Biodiversity

## Abstract

The study of freshwater fish species biodiversity and community composition is essential for understanding river systems, the effects of human activities on rivers, and the changes these animals face. Conducting this type of research requires quantitative information on fish abundance, ideally with long-term series and fish body measurements. This Data Descriptor presents a collection of 12 datasets containing a total of 146,342 occurrence records of 41 freshwater fish species sampled in 233 localities of various Iberian river basins. The datasets also contain 148,749 measurement records (length and weight) for these fish. Data were collected in different sampling campaigns (from 1992 to 2015). Eleven datasets represent large projects conducted over several years, and another combines small sampling campaigns. The Iberian Peninsula contains high fish biodiversity, with numerous endemic species threatened by various menaces, such as water extraction and invasive species. These data may support the development of large biodiversity conservation studies.

## Background & Summary

The Iberian Peninsula is considered one of the most biodiverse European regions and a fish biodiversity hotspot^1^. The Iberian freshwater fish fauna has the greatest European percentage of endemism (73% of the species) because of its long-term geographical isolation, which occurred during the last glaciation, the Mediterranean climate and the high number of different river basins^2^. This biodiversity is high at the species level but low at the family level, as most of the species belong to the family Cyprinidae^2^. Further, although local alpha diversity is low compared to that of tropical rivers, beta diversity, endemic species and threats make the Iberian rivers relevant ecosystems from the perspective of conservation^3^.

Iberian freshwater fish biodiversity is highly threatened: populations of 52% of the native species are under some degree of threat according to the International Union for the Conservation of Nature (IUCN)^4^. The main threat to Iberian freshwater fish is water extraction, which affects 60% of the native species, followed by introduced species (42% of the total Iberian freshwater fish richness), which affects 50% of the native species. Other important threats include climate change and pollution^4^.

The unique physical and biological characteristics and the long history of human activities make the Iberian Peninsula a very interesting place for the study of threats and conservation of freshwater fishes. However, Iberian freshwater fish species have received little attention^5^.

Gathering primary biodiversity data is necessary to improve our knowledge of the ecology, impacts and conservation status of freshwater fishes. Occurrence data are very useful for determining the distribution of species. However, distribution ranges are changing due to the diverse impacts caused by human activities. Accurately predicting the effects of human threats on communities and species requires more data. Time series of abundance data have proven essential for predicting population trends and assessing the risk of extinction of species^6^. If such data are accompanied by biological data, such as length and weight of individuals within a population, studies could be performed to analyse changes in population structure and dynamics caused by human impacts^7^. Models performed using these different data may prove more reliable for assessing human impacts and population trends, which would lead to better conservation and management plans for numerous species.

This Data Descriptor presents 12 different datasets of freshwater fish samplings in diverse locations of Spain performed by the Department of Environmental Biology of the University of Navarra in various rivers in Spain since 1992. Some of the studies have been completed, while others are open to further sampling campaigns in future years. In total, 146,342 occurrence records have been published to date, making this ichthyological collection one of the most important in Spain^8^, with the aim of offering the most complete information, both occurrence and measurement data, regarding the collected freshwater fish specimens.

## Methods

### Study area

Spain is the largest country of the Iberian Peninsula, located in southwestern Europe, delimited to the north by the Pyrenees and to the south by the Strait of Gibraltar. It is surrounded by the Mediterranean Sea to the East, the Cantabrian Sea to the north and the Atlantic Ocean to the West. The prevailing climate is Mediterranean, with hot, dry summers, rainy springs and autumns, and mild winters. Vegetation series in this climate are dominated by evergreen forests of holm oak (*Quercus ilex L*.) and shrubs (*Quercus coccifera* L., *Thymus vulgaris* L., *Rosmarinus officinalis* L. and others). In northern Spain, near the coast, the Oceanic climate predominates, with rain evenly distributed through the year, humid summers and mild winters. The vegetation there is dominated by deciduous forests of oak (*Quercus robur* L.) and beech (*Fagus sylvatica* L.). In the inland regions, these climates have continental and mountainous influences that create more extreme temperature variations.

These essential differences between climates shape and determine river ecosystems and species, creating four different freshwater ecoregions within the Iberian Peninsula^9^: the first includes the Cantabric Coast, with Oceanic climate. In this ecoregion, rivers are shorter and fast flowing through large mountains, with water all year. The second ecoregion is Eastern Iberia, which includes rivers that flow into the Mediterranean Sea (the Ebro, Ter and Júcar are the most important river basins). In this ecoregion, Mediterranean is the predominant climate, with continental characteristics in some areas. The third ecoregion is Western Iberia, which includes the Tagus and Duero river basins. These rivers flow to the Atlantic Sea through lands dominated by the Mediterranean climate. The last ecoregion is Southern Iberian; its most important basins are the Guadiana, Guadalquivir and Segura river basins. This ecoregion is dominated by the Mediterranean climate and includes the driest areas of the Iberian Peninsula. In the last three ecoregions, dominated by Mediterranean climate, rivers present high flow variability between seasons, with seasonal floods and droughts.

There are five main rivers in the Iberian Peninsula, the Ebro, Duero, Tajo, Guadiana and Guadalquivir, as well as numerous smaller basins. Due to this variability of climates, basins and habitats, the Iberian Peninsula has a high degree of freshwater fish biodiversity and endemism.

**Figure 1:**
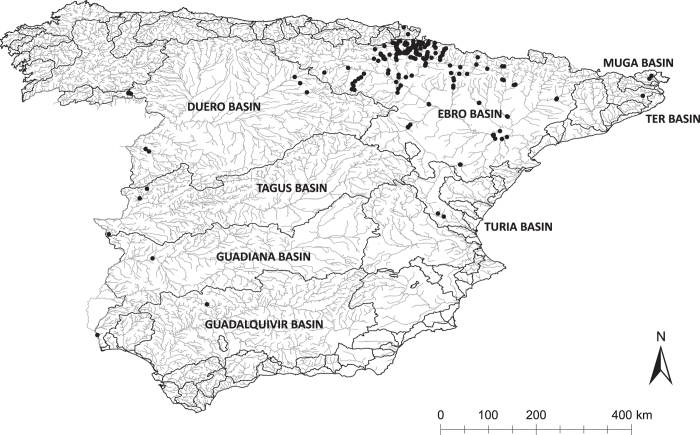
Map of Spain with the locations of all the sampling sites (black dots), the rivers (grey lines) and the river basins (black lines).

For this work, 233 localities of the Ebro, Duero, Tagus, Guadiana, Guadalquivir, Bidasoa, Ter, Muga and Turia river basins were sampled. They belong to eight Autonomous Communities and 15 provinces: Navarra, La Rioja, Catalonia (Lleida and Gerona), Aragon (Huesca, Zaragoza and Teruel), Castilla y León (Zamora, Burgos and Salamanca), Valencian Community (Valencia), Extremadura (Caceres and Badajoz) and Andalusia (Huelva and Córdoba). Most of the sampling locations (69%) and specimens (87%) were collected in Navarra, followed by Aragon, with 16% of the locations and 9% of the individuals. Eastern Iberia was the most sampled ecoregion, and the Ebro (Navarra, Aragon and La Rioja) was the most sampled river basin based on locations and specimens collected (Figure 1).

Samplings were conducted on dates during all seasons of the periods 1992–1998 and 2001-2015.

### Field sampling

In total, 148,812 specimens were collected by electrofishing, using an external generator (Honda EC3600) connected to an electrofishing control box and backpack electrofishing units (300–600 V, 0.2–2A). Two electrofishing methods were used, varying among projects: three-run depletion between two stop-nets; and semi-quantitative surveys, giving fish densities by catch per unit of effort (CPUE, number of specimens captured per hour)^10^. The sampling time of the semi-quantitative samplings varied between 15 min and 2 h, with 30 min being the most common sampling duration. The captured individuals were anaesthetized with tricaine methanesulfonate (MS-222; Sigma Chemical Co., St. Louis, MO) or 2-phenoxyethanol, identified, counted and measured (total length in millimetres, and, in some cases, weight in grams). Species recording and identification were performed by R. Miranda, J. Oscoz, P.M. Leunda, I. Vedia, I. Tobes, C. García-Fresca and A. Vilches using suitable literature^11,12^.

### Preservation

Once surveys were complete, fishes were returned to the river. For later studies, some captured specimens were euthanized with an overdose of anaesthesia, either preserved in jars with 70% ethyl alcohol or dried, and deposited in the Museum of Zoology of the University of Navarra. Each specimen was labelled with a unique collection number and introduced into the Museum database (Zootron v4.5 (ref. 13). In total, 2497 specimens from different datasets were preserved in jars or dried in the Museum (Table 1).

Then, datasets were exported to DarwinCore v1.4 format, revised, and corrected if necessary. Finally, Integrated Publishing Toolkit (IPT) resources for each dataset were created, metadata were added, and the Darwin Core Archives were uploaded. The resources were published in the Spanish Global Biodiversity Information Facility (GBIF) IPT node (http://www.gbif.es/ipt).

The Museum of Zoology of the University of Navarra (MZNA, Pamplona, Spain) has curated the scientific research materials of the Department of Environmental Biology since 1980. The Museum has provided data for the GBIF^14^.

## Data Records

Datasets include occurrence records and measurements (total length and weight) of captured fish. There are 146,342 occurrence records, representing 148,812 fish specimens. The collection comprises 13 families and 40 species of fishes (and one hybrid), belonging to the orders Anguilliformes, Cypriniformes, Siluriformes, Esociformes, Salmoniformes, Cyprinodontiformes, Scorpaeniformes and Perciformes. Cyprinidae is the most abundant family, with 25 species and 81% of the specimens. The other most abundant families are Salmonidae and Nemacheilidae, with 11 and 7% of the total specimens, respectively. Of the 40 species recorded, 20 are endemic to the Iberian Peninsula, and 14 are invasive. Moreover, 12 species are under some degree of threat according to the International Union for Conservation of Nature (IUCN)^15^: one is Critically Endangered, one Endangered, nine Vulnerable and one Near Threatened. Two species are not evaluated by the IUCN (Table 2).

Fish sampling data are split into 12 datasets with internal cohesion (, , , , , , , , , , , 12): six are part of PhD theses (, , , , , 6); five are multi-year funded projects (, , , , ) and another one () gathers several smaller samplings (Table 1). Datasets are accessible at the GBIF. All 12 datasets are in Darwin Core Archive format and have occurrence information for 35 Darwin Core terms: ocurrenceId, Modified, basisOfRecord, InstitutionCode, collectionCode, catalogNumber, Habitat, scientificName, kingdom, phylum, class, Order, Family, genus, specificepithet, taxonrank, scientificNameAuthorship, continent, country, stateProvince, locality, minimumElevationInMeters, maximumElevationInMeters, EVENTDATE, recordedBy, preparations, disposition, identifiedBy, verbatimEventdate, verbatimElevation, IndividualCount, decimalLongitude, decimalLatitude, geodeticDatum and verbatimCoordinates. They also include measurement information (total length and weight) of 9 Darwin Core elements: id, measurementID, measurementType, measurementValue, measurementAccuracy, measurementUnit, measurementDeterminedDate, measurementDeterminedBy and measurementRemarks.

Each GBIF resource contains a metadata section and the occurrence and measurements (length and weight) datasets in Darwin Core Archive format. Each resource has a different number of records and measurements, as well as a different type of records. Resources maintain an internal cohesion: project, river, studied species, etc. Three of the resources are ongoing projects and will be updated when necessary, whereas the other nine are finished projects (Table 1).

## Technical Validation

The two electrofishing techniques used are standardised and have been validated^16^. Species identification was performed using suitable literature^12^, and scientific names were validated using W. N. Eschmeyer’s Catalog of Fishes^17^. Before publication in GBIF, DARWIN_TEST application (v3.3 (ref. 18)) was used to eliminate possible mistakes in the coordinates, characters, taxonomy and the date format.

## Additional Information

**How to cite this article**: Rodeles, A. A. *et al.* Iberian fish records in the vertebrate collection of the Museum of Zoology of the University of Navarra. *Sci. Data* 3:160091 doi: 10.1038/sdata.2016.91 (2016).

## Supplementary Material



## Figures and Tables

**Table 1:** Summary of the main characteristics of the datasets.

**Dataset name**	**Field method**	**Ocurrence records**	**Measurement records (length)**	**Measurement records (weight)**	**Preserved records**	**Updates**	**Repository**	**Data link**
Fishes in MZNA-VERT: Freshwater communities in the Larraun river (Spain). PhD project, Javier Oscoz & Master project, A. Cos	Electrofishing	13,912	13,912	—	—	Closed dataset	GBIF	http://doi.org/10.15470/0qsajx
Fishes in MZNA-VERT: anatomy of cyprinids of Spain. PhD project, Rafael Miranda	Electrofishing	1249	1113	1112	1249	Closed dataset	GBIF	http://doi.org/10.15470/9nnmwv
Fishes in MZNA-VERT: Foraging ecology of the kingfisher. PhD project, Antonio Vilches	Electrofishing	21,868	21,864	—	10	Closed dataset	GBIF	http://doi.org/10.15470/wfpdpv
Fishes in MZNA-VERT: cyprinid and salmonid communities in the rivers Erro and Urederra (Spain). PhD project, Cristina García-Fresca	Electrofishing	27,033	27,008	—	338	Closed dataset	GBIF	http://doi.org/10.15470/bzoh5u
Fishes in MZNA-VERT: interactions between signal crayfish and fish communities. PhD project, Iván Vedia	Electrofishing	3032	3032	—	—	Closed dataset	GBIF	http://doi.org/10.15470/knqev7
Fishes in MZNA-VERT: freshwater populations in the Erro river (Spain). PhD Project, Pedro Leunda	Electrofishing	41,232	41,129	—	614	Closed dataset	GBIF	http://doi.org/10.15470/syfl1t
Fishes in MZNA-VERT: populations affected by the Itoiz dam in the Irati river (Spain)	Electrofishing	8567	8567	—	2	Closed dataset	GBIF	http://doi.org/10.15470/cefi2m
Fishes in MZNA-VERT: ecological assessment of the Aragon river in Sangüesa (Spain)	Electrofishing	3450	3450	—	—	Open dataset, updates when necessary	GBIF	http://doi.org/10.15470/msoj1m
Fishes in MZNA-VERT: distribution of freshwater blenny in the Segre and Susia rivers (Spain)	Electrofishing	3018	3014	142	6	Closed dataset	GBIF	http://doi.org/10.15470/d2ldy9
Fishes in MZNA-VERT: ecological assessment of the Guadalope river in Aliaga (Spain)	Electrofishing	432	432	—	—	Closed dataset	GBIF	http://doi.org/10.15470/sa3a33
Fishes in MZNA-VERT: monitoring program in the Suspiro stream (Spain)	Electrofishing	518	518	338	—	Open dataset, updates when necessary	GBIF	http://doi.org/10.15470/f1nnyp
Fishes in MZNA-VERT: baseline freshwater sampling campaigns	Electrofishing	22,031	21,969	1149	278	Open dataset, updates when necessary	GBIF	http://doi.org/10.15470/czwedx

**Table 2:** Species and specimens in the 12 datasets, along with family, IUCN category and zoogeographic origin data.

**Family**	**Species**	**n**	**IUCN category**	**Origin**
Anguillidae	*Anguilla anguilla*	35	Critically endangered A2bd+4bd	Native
Blenniidae	*Salaria fluviatilis*	1808	Least concern	Native
Centrarchidae	*Lepomis gibbosus*	25	Least concern	Introduced
	*Micropterus salmoides*	42	Least concern	Introduced
Cobitidae	*Cobitis calderoni*	216	Endangered A2ace+3ce	Endemic
	*Cobitis paludica*	4	Vulnerable A2ce+3ce	Endemic
Cottidae	*Cottus aturi*	137	Least concern	Native
Cyprinidae	*Achondrostoma arcasii*	1981	Vulnerable A3ce	Endemic
	*Alburnus alburnus*	2617	Least concern	Introduced
	*Barbus comizo x Luciobarbus microcephalus*	3		Endemic
	*Barbus haasi*	763	Vulnerable A2ce+3ce	Endemic
	*Barbus meridionalis*	53	Near threatened	Native
	*Carassius auratus*	85	Least concern	Introduced
	*Cyprinus carpio*	190	Least concern	Introduced
	*Gobio lozanoi*	8815	Least concern	Endemic
	*Iberochondrostoma lemmingii*	31	Vulnerable A2ace+3ce	Endemic
	*Luciobarbus bocagei*	171	Least concern	Endemic
	*Luciobarbus comizo*	31	Vulnerable A2ce	Endemic
	*Luciobarbus graellsii*	15,401	Least concern	Endemic
	*Luciobarbus guiraonis*	37	Vulnerable A3ce	Endemic
	*Luciobarbus microcephalus*	42	Vulnerable A2ce+3ce	Endemic
	*Luciobarbus sclateri*	85	Least concern	Endemic
	*Parachondrostoma miegii*	28,861	Least concern	Endemic
	*Phoxinus bigerri*	59,191	Least concern	Native
	*Pseudochondrostoma polylepis*	213	Least concern	Endemic
	*Rutilus rutilus*	172	Least concern	Introduced
	*Scardinius erythrophthalmus*	51	Least concern	Introduced
	*Squalius alburnoides*	249	Vulnerable A3ce	Endemic
	*Squalius carolitertii*	34	Least concern	Endemic
	*Squalius laietanus*	216	Least concern	Endemic
	*Squalius pyrenaicus*	92	Not evaluated	Endemic
	*Squalius valentinus*	3	Vulnerable B1ab	Endemic
	*Tinca tinca*	66	Least concern	Introduced
Esocidae	*Esox lucius*	5	Least concern	Introduced
Ictaluridae	*Ameiurus melas*	207	Least concern	Introduced
Nemacheilidae	*Barbatula quignardi*	10,780	Least concern	Endemic
Percidae	*Sander lucioperca*	33	Least concern	Introduced
Poeciliidae	*Gambusia holbrooki*	6	Least concern	Introduced
Salmonidae	*Oncorhynchus mykiss*	130	Not evaluated	Introduced
	*Salmo trutta*	15,924	Least concern	Native
Siluridae	*Silurus glanis*	7	Least concern	Introduced
